# Prescribing trends and rational drug use patterns in cardiovascular patients: A cross-sectional observational study

**DOI:** 10.6026/9732063002001582

**Published:** 2024-11-30

**Authors:** Radhika Chikatipalli, Anitha Kuttiappan, Sanjeev Kumar, Vishali R., Priyanka Kujur, Afsal N.A, Poojitha N.R, Santenna Chenchula

**Affiliations:** 1 Department of Pharmacology, Sri Venkateswara College of Pharmacy, Chittoor, India; 2 SVKM'S NMIMS, School of Pharmacy and Technology Management, Shirpur, India; 3 Department of Pharmacology, People's College of Medical Sciences & Research Centre, Bhopal, India; 4 Department of Pharmacology, All India Institute of Medical Sciences, Bhopal, India

**Keywords:** Cardiovascular diseases, rational drug use, prescription pattern analysis, essential drug list, drug utilization, WHO core drug use indicators

## Abstract

Cardiovascular diseases (CVDs), encompassing conditions like coronary artery disease, hypertension and ischemic heart disease, are highly prevalent worldwide. This study analyzed prescribing trends and treatment appropriateness in CVD patients, focusing on adherence to guidelines, essential medicine use and generic drug prescriptions. A cross-sectional observational study was conducted on CVD-diagnosed patients. Data on prescribed medications-including drug classes, generic prescriptions and adherence to the Essential Drug List (EDL)-were collected and analyzed using IBM SPSS version 26.0. The most frequently prescribed drug classes included antiplatelets, diuretics and hypolipidemics. Aspirin was the most commonly prescribed medication (58.1%), followed by furosemide (36.5%), amlodipine (32.4%) and rosuvastatin (24.3%). Statins and calcium channel blockers were prescribed more often than angiotensin II receptor blockers and beta-blockers. On average, 13.2 drugs were prescribed per patient, with only 28.8% prescribed generically. Furthermore, 47.3% of medications were on the EDL. This study highlights the high prevalence of CVDs and the common drug classes prescribed to manage them. These findings provide important insights into current prescribing trends, particularly the frequent use of anti-hypertensive, antiplatelets, diuretics and hypolipidemics and suggest areas for optimizing medication management in this population. Additionally, there is a need to better manage polypharmacy in CVD patients.

## Background:

Cardiovascular diseases (CVDs) collectively remain the leading cause of global mortality, significantly contributing to the deterioration of health and imposing substantial costs on health systems [1]. In 2020, CVDs accounted for approximately 19.1 million deaths worldwide, with an age-adjusted death rate of 239.8 per 100,000 people and an age-adjusted incidence rate of 7354.1 per 100,000 people [1-2]. Notably, Eastern Europe and Central Asia experienced the highest mortality rates related to CVD incidence [2]. Low- and middle-income countries bear the brunt of CVD deaths, with nearly a quarter of all deaths in India attributed to CVDs [3]. The World Health Organization (WHO) estimates that India may incur a loss of $237 billion over the next decade due to reduced productivity and increased healthcare spending [1]. Specifically, coronary artery disease (CAD) and stroke constitute 83% of CVD mortality in India, with CAD disproportionately affecting younger age groups [4]. Prescription Pattern Monitoring Studies (PPMS) play a crucial role in drug utilization research, focusing on prescribing, dispensing and administering drugs [5]. These studies aim to encourage appropriate drug use, mitigate abuse or misuse and enhance understanding of drug utilization trends, drug quality, compliance with treatment guidelines, usage of essential medicines and adoption of generic drugs [5].

Studying prescription patterns is essential for optimizing drug therapy, resource utilization and reducing prescription errors [6]. The WHO reports that more than half of all medicines are dispensed or sold inappropriately, with half of patients failing to adhere to correct medication usage [6]. The rational use of medicines, as defined by the WHO, emphasizes the importance of patients receiving medications suitable for their clinical needs, in appropriate doses, for adequate durations and at minimal cost to both the patient and the community [7]. Irrational prescribing practices can lead to unsafe and ineffective treatment, exacerbate or prolong disease states, cause harm and distress to patients and increase overall healthcare costs [7]. The WHO's validated first-line drug use indicator is crucial for assessing drug use patterns [7]. These indicators, such as the average number of drugs per encounter, the percentage of drugs prescribed using generic names, the percentage of encounters with prescribed antibiotics or injections and the percentage of drugs prescribed from the essential drug list, offer informative insights that are less likely to fluctuate over time and place [8]. Therefore, it is of interest to assess the prescribing trends and treatment appropriateness in CVD patients using WHO Core drug use indicators.

## Methods:

This cross-sectional, observational study was designed to assess prescription drug patterns among patients diagnosed with cardiovascular disease (CVD) admitted to the Cardiac Intensive Care Unit (CICU) and general ward of a tertiary care hospital in Bengaluru, India.

## Ethical considerations:

The study protocol received approval from the Institutional Ethics Committee (IEC) (Ref. No: IEC/RVSIMS/2022/01). Confidentiality was strictly maintained throughout the study, with data used exclusively for research purposes. The study was conducted over a six-month period following ethics approval.

## Inclusion criteria:

The study included patients aged 18 years or older who were diagnosed with CVD, with or without comorbid conditions. Exclusion criteria were pregnant or lactating women, children below 18 years of age, individuals with unclear diagnoses and records with incomplete data.

## Study methodology:

A total of 230 case records of CVD patients were reviewed during the study period. Physicians followed their usual prescribing practices without any intervention from the research team. Data collected from the case records included demographic details (age, sex and date of admission), presenting symptoms, medical history, vital signs, physical examination results, electrocardiogram (ECG) findings, laboratory results (lipid profile, complete blood count [CBC]), comorbidities and prescription information (drug names, whether generic or brand, dosage, form and frequency). The methodology adhered to established protocols for prescription pattern analysis [5, 8, 13 and 17].

## Statistical analysis:

Descriptive statistics were used to summarize the data, with categorical variables presented as frequencies and percentages. Data analysis was performed using SPSS version 26.0.

## Results:

## Demographic profile:

A total of 230 prescriptions were analyzed. Of the patients diagnosed with CVD, 151 (65.65%) were male, while 79 (34.34%) were female. The age distribution indicated that the majority of cases occurred in patients aged over 61 years, highlighting an age-related predisposition for CVD.

## Prevalence of cardiovascular diseases:

Among the study population, coronary artery disease (CAD) was the most frequently diagnosed cardiovascular condition, observed in 19.56% of the patients. Hypertension was present in 18.26% of patients, followed by ischemic heart disease, which accounted for 13.47% of cases.

## Comorbidities:

The most prevalent comorbidity in the study population was hypertension, affecting 41.73% of patients, followed by diabetes mellitus, observed in 23.91% of patients (Table 2).

## Prescription patterns:

On average, each patient was prescribed 13.19 drugs (Table 1). Aspirin was the most commonly prescribed cardiovascular drug, appearing in 58.10% of prescriptions, followed by furosemide (36.48%), amlodipine (32.43%) and rosuvastatin (24.32%). Among the drug classes, statins and calcium channel blockers were prescribed more frequently than angiotensin II receptor blockers and beta-blockers. Rosuvastatin was the most common statin, while metoprolol was the most widely used beta-blocker. Amlodipine (32.43%) was the most prescribed calcium channel blocker and heparin (17.56%) was the most frequently used anticoagulant.

## Other prescribed medications:

In addition to cardiovascular drugs, patients were prescribed various medications for symptomatic relief or the management of underlying conditions. Antiulcer agents were prescribed to 79.56% of patients, with pantoprazole being the most common. Antibiotics were prescribed in 75.08% of cases, with ceftriaxone and azithromycin being the most frequently used. Insulin was administered to 45.65% of patients and other oral hypoglycemic agents, such as metformin (3.04%) and glimepiride (2.17%), were also prescribed. Commonly used analgesics and antipyretics included paracetamol (23%) and tramadol (5.21%).

## Who drug prescribing indicator:

The analysis of prescription patterns based on WHO drug prescribing indicators, as shown in Table 3, revealed several key findings. On average, 13.90 drugs were prescribed per patient encounter, indicating a high volume of medication use. Antibiotics were included in 76.08% of the prescriptions, highlighting their widespread use in the study population. Approximately 47.28% of the prescribed drugs were from the 2022 Essential Drug List (EDL), reflecting adherence to standard treatment guidelines. However, only 28.83% of the medications were prescribed by their generic names, while the majority (71.16%) were prescribed by brand names, suggesting a preference for branded medications in clinical practice. Furthermore, 34% of the drugs were administered via injection, indicating a significant reliance on parenteral therapy. These findings provide valuable insights into the prescription trends in this healthcare setting, emphasizing both the areas of guideline adherence and the potential for optimization in drug prescribing practices.

## Discussion:

This cross-sectional observational study evaluated the prescription drug patterns in patients with cardiovascular diseases (CVDs) admitted to the Cardiac Intensive Care Unit (CICU) and general ward of a tertiary care hospital in India. Based on data from the National Health and Nutrition Examination Survey (NHANES) 2017 to March 2020, the overall incidence of CVD-including coronary heart disease (CHD), heart failure (HF), stroke and hypertension-among adults aged ≥20 years was 48.6% (127.9 million in 2020), with an increasing incidence of CVD with age in both males and females. According to 2020 mortality data, heart diseases and stroke were responsible for more annual deaths than cancer and chronic lower respiratory diseases combined. In 2020, the mortality rate from heart disease and stroke was 207.1 per 100,000 people. Globally, an estimated 19.05 million deaths were attributed to CVD in 2020, reflecting an 18.71% increase since 2010. The age-standardized death rate per 100,000 people was 239.80, a 12.19% decrease compared to 2010 [9]. Drug utilization studies have been conducted worldwide across various healthcare settings to assess prescription patterns and address the irrational use of medications [10]. Coronary artery disease (CAD) remains the leading single cause of mortality and loss of disability-adjusted life years (DALYs) globally, with a particularly high burden in low- and middle-income countries. CAD accounts for nearly 7 million deaths and 129 million DALYs annually, posing a significant economic challenge [11]. In our study, 19.56% of patients were diagnosed with CAD, followed by hypertension (18.26%) and ischemic heart disease (13.47%). Among CAD patients, diagnoses included triple vessel disease (TVD), single vessel disease (SVD) and double vessel disease (DVD), with some cases identified solely as CAD. The prevalence of myocardial infarction (MI), including both anterior wall MI (AWMI) and inferior wall MI (IWMI), was 10%, with ST-elevation MI (STEMI) and non-ST-elevation MI (NSTEMI) accounting for 3.47% and 7.39% of cases, respectively. Angina was reported in approximately 7.82% of cases, consistent with findings from Saranya *et al.* (10.4%) [12], additionally arrhythmias were observed in 5.65% of cases, aligning with the 3.3% reported by Abdul *et al.* [13].

Comorbid conditions were prevalent in the study population, with hypertension combined with type 2 diabetes mellitus present in 41.73% of patients, while 15.65% had diabetes mellitus alone, consistent with findings from Belhekar *et al.* and Abdul Hanan *et al.* [13, 14]. Among the 230 participants, the male-tofemale ratio was 65.65% to 34.34% and CVD incidence was highest in patients aged 66 years and older (55.65%), corroborating findings from other studies [15, 16, 17-18]. Regarding drug prescriptions, antiplatelet agents (93.91%), hypolipidemic agents (61.73%), and diuretics (61.73%) were the most commonly prescribed cardiovascular drugs. These results are comparable to those reported in other studies [19, 20, 21, 22, 23, 24, 25-26]. Aspirin was prescribed to 66.08% of patients, and dual antiplatelet therapy (aspirin + clopidogrel) was given to 3.47%, in accordance with recommendations by the Association of Physicians of India for myocardial infarction (MI) cases [26]. The most frequently prescribed hypolipidemic agents were rosuvastatin (31.73%) and atorvastatin (27.39%), although the overall use of these agents was lower compared to other studies [16, 19, 20-21]. Diuretics such as spironolactone (23.47%) and furosemide (38.26%) were prescribed to 61.73% of patients. Beta-blockers and calcium channel blockers were used in 36.52% and 25.65% of patients, respectively, with metoprolol and carvedilol being the most commonly prescribed beta-blockers, and amlodipine the most frequently prescribed calcium channel blocker. Telmisartan was the most commonly used angiotensin II receptor blocker (ARB) (19.13%). These findings differ from those of Vakade *et al.* where ACE inhibitors and ARBs were more frequently prescribed than beta-blockers and calcium channel blockers [24, 25- 26, 27].

A study at Gondar University Specialized Hospital in Ethiopia assessed prescribing trends and their impact on clinical outcomes in 833 cardiovascular patients, most of whom were female (62.5%) and over 50 years old (61%). Diuretics were the most commonly prescribed drugs, either as monotherapy (33.6%) or in combination with angiotensin-converting enzyme (ACE) inhibitors (21.8%) or calcium channel blockers (8.3%). Over a 72-month follow-up, combination therapies were associated with significantly better clinical improvement (Log Rank = 28.9, P = 0.000). Despite these positive outcomes, the study noted a limited range of prescribed cardiovascular drugs, suggesting the need for more diverse prescribing practices to enhance patient care [28].

A study of 574 coronary care unit (CCU) patients revealed that most were male (65%) and under 60 years old (57%), with 72.6% diagnosed with coronary artery disease (CAD). Commonly prescribed drug classes included platelet inhibitors (88.7%), statins (76.3%), ACE inhibitors/angiotensin receptor blockers (72%), beta-blockers (58%) and heparin (57%), with poly-pharmacy (>5 drugs) observed in 71% of patients. CAD patients received more medications and had longer CCU stays (p < 0.0001). Clinical comorbidities, such as renal dysfunction, ST-elevation myocardial infarction and bradyarrhythmias, were associated with reduced use of ACE inhibitors, calcium channel blockers and beta-blockers, respectively. The findings highlight frequent poly-pharmacy and the impact of clinical conditions on cardiovascular drug utilization [29].

The most common inotropic agents were noradrenaline, followed by dobutamine and dopamine, differing from studies by Fardan *et al.* and Nagabushan *et al.* where dopamine was the predominant inotrope [15, 21]. This variation highlights the differences in inotrope use across cardiovascular patient populations. In addition to antibiotics (76.08%), other commonly prescribed miscellaneous drugs included anti-ulcer agents, anti-emetics, stool softeners and anxiolytics. Pantoprazole (22.17%) was the most frequently used proton pump inhibitor, while ondansetron was the primary antiemetic. Paracetamol (23%) was the most commonly used non-steroidal anti-inflammatory drug (NSAID) and opioid analgesics, such as tramadol and fentanyl (10.86%), were in line with findings by Barot *et al.* [25]. Lactulose (14.78%) was the most commonly prescribed stool softener and alprazolam (19.56%) the most frequently used anxiolytic, aligning with the findings of Vakade *et al.* [27]. The average number of drugs prescribed per patient was 13.19, indicating a high prevalence of polypharmacy compared to other studies [15, 16]. Only 28.83% of drugs were prescribed in their generic form, suggesting a need for improvement in this area to enhance cost-effectiveness. Antibiotic prescriptions were high, with 76.08% of encounters including antibiotics, surpassing the rates reported by Chandana *et al.* (25%) and Chandana *et al.* (19.5%) [30, 31]. Drugs prescribed via injection accounted for 30.34%, with cephalosporin (ceftriaxone) being the most common antibiotic, consistent with findings from Saranya *et al.* [12]. Moreover, 47.98% of prescribed drugs were from the Essential Drug List, similar to findings from global studies [32, 33 34, 35, 36- 37]. Overall, the study points to both strengths and areas for improvement in prescription practices, particularly in reducing polypharmacy, enhancing adherence to guidelines, increasing the use of generic medications and optimizing antibiotic and injection use. These findings underscore the need for targeted interventions to ensure rational drug use in cardiovascular patients and enhance the quality of care in this population.

## Conclusion:

This study provides important insights into prescribing patterns for CVD patients, with a notable emphasis on the frequent use of anti-hypertensives, anti-platelets, diuretics and hypolipidemics. While most medications were prescribed in line with current treatment guidelines, the high reliance on brand-name drugs highlights the need to increase generic prescribing to reduce patient costs. Furthermore, the high incidence of polypharmacy underscores the necessity of implementing screening tools such as the Beers criteria or STOPP/START criteria to optimize medication use and minimize unnecessary drug exposure. Additionally, efforts should focus on prescribing drugs from the Essential Drug List (EDL) to further enhance clinical practice and patient care.

## List of abbreviations:

CVDs: Cardiovascular diseases

CICU: Cardiac Intensive Care Unit

EDL: Essential Drug List

WHO: The World Health Organization

CAD: Coronary artery disease

PPMS: Prescription Pattern Monitoring Studies

NHANES: The National Health and Nutrition Examination Survey

DALYs: Disability-adjusted life years

AWMI: Anterior wall myocardial infarction

STEMI: ST-elevation myocardial infarction

NSTEMI: Non-ST-elevation myocardial infarction

NSAIDs: Non-steroidal anti-inflammatory drugs

## Ethics approval:

The study was approved by the Institutional Ethics Committee at the RVS Institute of Medical Sciences, Chittoor and Andhra Pradesh, India. (Ref. No.: IEC/RVSIMS/2022/01).

## Availability of data and material:

All data generated or analyzed during this study are included in this article.

## Authors' contributions:

RV, PK, ANA and KRP designed and conducted the study. RC, AK, SK and CS: Supervised the work, wrote and reviewed the manuscript and finalized the manuscript.

## Figures and Tables

**Table 1:** Percentage of cardiovascular drug prescribed in the study population

**Class**	**Drugs**	**Percentage**	**Total**
Anti-platelets	Aspirin	66.08%	152
	Clopidogrel	24.34%	56
	Aspirin+clopidogrel	3.48%	8
	Ticagrelor	23.04%	53
Diuretics	Spirolactone	23.47%	54
	Furosemide	38.26%	88
Statins	Rosuvastatin	31.73%	73
	Storvastatin	2.61%	6
	Atorvastatin	27.39%	63
Anti-coagulants	Enoxaparin	9.57%	22
	HEPARIN	15.21%	35
Angiotensin-ii receptor blockers	Telmisartan	19.13%	44
Calcium channel blocker	Amlodipine	25.65%	59
B-blockers	Metoprolol	15.21%	35
	Carvedilol	21.30%	49
Ace inhibitor	Ramipril	2.61%	6
Anti-anginal agents	Trimetazidine	10.86%	25
	RANOLAZINE	4.35%	10
Antiarrhythmics	Amiodarone	13.91%	32
Antidiabetics	Human actrapid	45.65%	105
Vasodilators	Nitrate	7.83%	18
B1/β2 ADRENERGIC AGENTS	Isoprenaline	1.30%	3
A-blockers	Clonidine	2.61%	6
Inotropes	Dopamine	2.17%	5
	Dobutamine	4.34%	10
	Noradrenaline	5.21%	12

**Table 2:** Percentage of miscellaneous drug prescribed

**S No.**	**Miscellaneous drugs**	**No. of drugs**	**Percentage**
1	Anti-biotics	175	76.08%
2	Anti-ulcer	183	79.56%
3	Anti-emetics	51	22.17%
4	Anxiolytics	92	40%
5	Thyroid Agent	14	6.08%
6	Anti-diabetics	16	52%
7	Laxative	34	14.78%
8	Analgesics	78	33.91%
9	Insulin	105	45.65%

**Table 3:** WHO core drug prescribing indicator

**S No.**	**Drug use indicators**	**Results**
1	Total no. of drugs prescribed	3035
2	Total no. of encounters	230
3	Average no. of drugs per encounter	13.19
4	Percentage of prescriptions with injections	30.34%
5	Percentage of prescriptions with antibiotics	76.08%
6	Percentage of drugs prescribed by generic name	28.83%
7	Drugs prescribed from the essential drug list	47.28%
